# Anterior cervical decompression and fusion for Samartzis Type I Klippel–Feil syndrome complicated by cervical radiculopathy with radiographic spinal cord compression: a case report of sandwich-type interposed mobile segment

**DOI:** 10.3389/fsurg.2026.1888847

**Published:** 2026-09-24

**Authors:** Baopeng Zeng, Shengxing Liu

**Affiliations:** Department of Spine Surgery, The Second Affiliated Hospital of Hainan Medical University, Haikou, China

**Keywords:** adjacent segment degeneration, anterior cervical discectomy and fusion, biomechanics, case report, cervical radiculopathy, Klippel-Feil syndrome, Samartzis classification, sandwich- type interposed mobile segment

## Abstract

**Background:**

Klippel-Feil syndrome (KFS) is a rare congenital cervical malformation caused by embryonic segmentation defects leading to vertebral fusion, which alters cervical biomechanics and predisposes to premature spinal degeneration. The “sandwich-type interposed mobile segment” refers to a solitary mobile cervical segment trapped between congenital bony fusion and iatrogenic surgical fusion. It bears concentrated mechanical stress and carries a high risk of early adjacent segment degeneration (ASD). Current epidemiological data for postoperative ASD are mostly derived from general spinal cohorts, whereas targeted clinical evidence for KFS patients remains limited. This study systematically retrieved KFS cervical surgical cases from Web of Science and PubMed to explore clinical features and long-term degenerative risks of this unique biomechanical lesion.

**Case presentation:**

A 44-year-old female with Samartzis Type I KFS presented with intractable cervical radiculopathy and radiographic spinal cord compression. After 6 months of failed conservative management, she underwent C6/7 anterior cervical discectomy and fusion (ACDF). Radicular symptoms resolved immediately, with full neurological recovery at the 12-month follow-up. Symptomatic C5/6 ASD occurred 40 months postoperatively. Revision ACDF was performed after repeated conservative treatment failure, and complete neurological remission was achieved 6 months after revision. No extra-spinal congenital anomalies were identified. The C5/6 level represented the only functional mobile segment sandwiched between congenital C4/5 fusion and iatrogenic C6/7 fusion, forming a high-risk stress-concentrating biomechanical construct.

**Conclusion:**

ACDF yields favorable shortterm outcomes for Samartzis Type I KFS patients with cervical radiculopathy and spinal cord compression. Nevertheless, KFS patients with sandwich-type interposed mobile segments may face an increased risk of early symptomatic ASD. Combined with nine retrieved KFS surgical cases, our findings suggest the necessity of individualized surgical planning and lifelong imaging surveillance owing to persistent biomechanical abnormalities. This case supplements scarce clinical and biomechanical evidence for KFS related early postoperative degeneration and provides references for preoperative risk stratification in similar clinical scenarios.

## 1 Introduction

Klippel-Feil syndrome (KFS) is a rare congenital developmental disorder characterized by fusion of two or more cervical vertebrae caused by embryonic segmental dysplasia, with an incidence of 0.05%–0.3% in the general population (1–3).

The disease occurs sporadically in most clinical cases (4–6), with no obvious gender predominance, and the classic triad of short neck, low posterior hairline, and limited cervical motion is only observed in a minority of patients (7, 8). Most cases remain clinically silent until secondary cervical degeneration induces neurological symptoms, resulting in delayed diagnosis and intervention (3, 9).

The Samartzis classification, the most authoritative grading system for KFS, divides the disease into three subtypes according to fusion characteristics (8, 10).

Type I (the subtype of the present patient) refers to single isolated congenital fusion; Type II presents multiple discontinuous fused segments; Type III manifests continuous multi-level cervical fusion. Different subtypes lead to distinct cervical biomechanical states and long-term degenerative risks (9, 10). Congenital vertebral fusion destroys normal cervical stress conduction, concentrating excessive mechanical load on residual mobile segments, thereby accelerating premature disc degeneration, facet joint hyperplasia, spinal stenosis, and nerve root or spinal cord compression (9, 11). Chronic neck pain and upper limb neurological symptoms are the most common clinical manifestations in symptomatic KFS patients, severely affecting patients’ quality of life (12).

In general cervical spondylosis cohorts, the incidence of radiographic ASD after ACDF is approximately 30%, while the incidence of symptomatic ASD requiring clinical intervention is about 11%, with a 10-year cumulative reoperation rate of 10.4% (13–15).

However, these epidemiological benchmarks are all derived from ordinary populations without congenital spinal malformations (13, 16). After systematic retrieval of Web of Science Core Collection and PubMed databases (strictly following case report writing and submission specifications), a total of 9 valid KFS cervical spine surgical cases were included in this study, covering multiple surgical modalities including ACDF, cervical disc arthroplasty (CDA), posterior decompression, and customized internal fixation, as well as special clinical scenarios such as combined Sprengel deformity and rare postoperative complications (17–21). Our literature review indicates that existing KFS surgical studies are mostly scattered single-case reports, lacking systematic biomechanical analysis and long-term outcome summary, and there is no unified consensus on surgical modality selection and ASD risk prevention for KFS patients with special sandwich-type anatomical structure.

At present, the selection of surgical strategies for KFS remains controversial. CDA can theoretically preserve segmental mobility and reduce ASD risk in general cervical populations (22, 23), but evidence regarding its safety and efficacy in KFS patients with congenital biomechanical imbalance remains scarce, and its long-term effectiveness in this patient population has not been established (24). Among the retrieved KFS surgical cases, CDA has only been reported in a small number of young patients with simple single-segment lesions, whether prosthesis-related complications or abnormal stress distribution would occur in patients with multi-level fusion or special anatomical malformations remains unclear due to limited clinical data (24). By contrast, ACDF can achieve reliable spinal stability and is the mainstream surgical method for symptomatic KFS patients with spinal cord and nerve root compression (9, 25).

For KFS patients, systematic multi-system malformation screening is essential before surgery, as the disease is often combined with visceral and musculoskeletal malformations involving the heart, kidney, and auditory system (6, 7, 11).

For asymptomatic KFS patients, regular imaging follow-up is the main management strategy; for patients with mild symptoms, standardized conservative treatment including physical therapy, drug intervention, and cervical immobilization is preferred. Surgical decompression and fusion are indicated for patients with progressive neurological dysfunction and intractable severe pain (9, 12, 18).

## 2 Materials and methods

### 2.1 Literature retrieval and screening strategy

#### 2.1.1 Database selection

This study is a rare disease clinical case report, complying with the submission specifications of mainstream spinal surgery journals.

Two core biomedical databases, Web of Science Core Collection and PubMed, were selected for literature retrieval. PubMed comprehensively covers clinical case reports and small-sample case series, while Web of Science Core Collection includes high-quality authoritative studies in high-impact journals. The dual-database combination ensures the comprehensiveness and academic credibility of retrieved literature, which is the standard retrieval scheme for clinical case report studies and does not require additional retrieval of systematic review-specific databases such as Embase and Cochrane.

#### 2.1.2 Retrieval strategy

The retrieval themes focused on Klippel-Feil syndrome, congenital cervical fusion, cervical spondylosis, cervical radiculopathy, spinal cord compression, and cervical spine surgery.

A combination of MeSH subject terms and free words was adopted, with no restrictions on publication time, region, or language. Web of Science adopted TS (topic field: title, abstract, keywords) retrieval to avoid full-record retrieval noise and prevent omission of rare disease literature. PubMed adopted MeSH term + free word combined retrieval, and valid case studies were screened through the built-in database literature type filter after retrieval.

#### 2.1.3 Inclusion and exclusion criteria

Inclusion criteria: (1) patients definitely diagnosed with Klippel-Feil syndrome; (2) complicated with cervical degenerative diseases, cervical radiculopathy or myelopathy with radiographic neural compression; (3) received cervical spine surgical intervention; (4) studies were case reports or case series with complete clinical, imaging and follow-up data.

Exclusion criteria: (1) reviews, meta-analyses, basic experimental studies and epidemiological research without individual case data; (2) KFS patients treated with simple conservative therapy without surgical intervention; (3) repeatedly published, incomplete or low-quality literature with unavailable valid data.

#### 2.1.4 Screening results

After dual-database retrieval, manual deduplication, title-abstract preliminary screening and full-text secondary screening, a total of 9 valid KFS cervical surgical case studies were finally included, including 2 high-quality cases from Web of Science and 7 clinical cases from PubMed.

The included studies cover diverse surgical methods and complex clinical scenarios, forming a comprehensive literature basis for the comparative analysis of the present case. Basic information of all included cases is summarized in Table 2 (19–21, 24–29).

### 2.2 Clinical data

A 44-year-old female patient was admitted to our hospital due to persistent neck pain accompanied by left upper limb radiating pain for 6 months, with aggravated left hand weakness for 2 weeks.

The patient had no history of cervical trauma, prior cervical surgery, familial hereditary disease or malignant tumor. She had received standardized conservative treatment including physical therapy, anti-inflammatory and analgesic drugs, and cervical immobilization for 6 months, but her symptoms continued to progress without sustained improvement (Table 1).

**Table 1:** Standardized clinical timeline.

Time Node	Clinical Event
6 months before first admission	Onset of neck pain and left upper limb radiating pain
2 weeks before hospitalization	Progressive left hand weakness; standardized conservative therapy ineffective
Admission day 1	Complete physical examination + cervical x-ray, 3D-CT, preoperative MRI + multi-system malformation screening
Hospitalization week 2	Elective C6/7 ACDF surgery
Postoperative 12 months	Complete relief of neurological symptoms; solid bony fusion confirmed by imaging
Postoperative 40 months	Recurrent bilateral upper limb numbness and fatigue; MRI confirmed C5/6 disc herniation and spinal stenosis (symptomatic ASD)
1 month after symptom recurrence	Repeated conservative treatment failed
External hospital admission	C5/6 revision ACDF was performed; postoperative follow-up imaging studies were obtained
Revision surgery 6-month follow-up	Full resolution of clinical symptoms; stable internal fixation and solid fusion verified by CT

**Table 2:** Basic information of included 9 Klippel-Feil syndrome surgical cases.

No.	Author & year	Database	KFS Subtype/combined malformation	Surgical procedure	Main clinical outcomes & complications
1	Yi et al., 2007 (24)	PubMed	Single-level congenital fusion, no combined malformation	Cervical disc arthroplasty (CDA)	Short-term neurological improvement; potential abnormal segmental stress concentration, suggesting limited applicability of CDA in KFS patients
2	Suzuki et al., 2008 (28)	PubMed	Multi-level fusion, atlantoaxial pseudoarthrosis	Posterior cervical decompression	Relieved myelopathy symptoms; high risk of intraoperative bony structure injury in KFS combined with spinal malformation
3	Füllbier et al., 2010 (19)	PubMed	Combined Sprengel deformity & omovertebral bone	Posterior decompression and osteotomy	Complete spinal cord decompression achieved; individualized osteotomy required for combined skeletal malformations
4	Mahajan et al., 2021 (27)	PubMed	Multi-level irregular bony fusion malformation	Anterior cervical fusion	Solid fusion achieved; confirmed abnormal bony growth and high degenerative risk in KFS
5	Parish et al., 2021 (20)	PubMed	Sprengel deformity with canal-occupying omovertebral bone	Precision posterior decompression	Progressive myelopathy completely relieved; emphasized preoperative individualized anatomical evaluation
6	Pompliano et al., 2021 (21)	PubMed	Sprengel deformity + traumatic spinal cord compression	Emergency anterior decompression & fusion	Neurological function recovered gradually; KFS patients are more vulnerable to traumatic spinal injury
7	Jackson et al., 2022 (25)	Web of Science	Complex multi-level fusion deformity	Patient-specific custom plate fixation	Stable spinal reconstruction achieved; verified the necessity of customized surgery for severe KFS malformation
8	Chatzikomninos et al., 2024 (26)	PubMed	Common-type KFS without combined malformation	Anterior cervical decompression & fusion	Rare postoperative white cord syndrome occurred; indicating an elevated risk of neurological complications after cervical surgery in KFS
9	Nie et al., 2023 (29)	Web of Science	Severe multi-level cervical fusion & spinal stenosis	Transmandibular cervical corpectomy	Thorough decompression achieved; special surgical approach is required for severe KFS with complex compression

### 2.3 Ethical statement

Written informed consent was obtained from the patient for the publication of this case report and all associated imaging images.

All radiological data were desensitized to remove personally identifiable information. This case report study does not involve invasive experimental interventions and was approved for ethical exemption by the institutional review board.

### 2.4 Physical examination and auxiliary screening

The patient was conscious with significantly restricted cervical range of motion. Neurological examination showed decreased pain and temperature sensation in the left middle and ring fingers, left hand muscle strength grade 3, impaired fine finger movement, and diminished left triceps reflex. Bilateral Hoffmann signs and lower limb pathological reflexes were negative. Systemic auxiliary examinations including renal ultrasound, electrocardiogram, audiometry and full-spine radiography excluded extra-cervical multi-system congenital malformations associated with KFS.

### 2.5 Imaging findings

Cervical flexion-extension radiographs showed intact cervical alignment without dynamic instability.

Cervical 3D-CT confirmed congenital osseous fusion of C4/5 vertebrae, consistent with Samartzis Type I KFS. Preoperative cervical MRI revealed left posterior C6/7 disc herniation, secondary spinal stenosis, and obvious ventral spinal cord and left nerve root compression (Figure 1).

**Figure 1:**
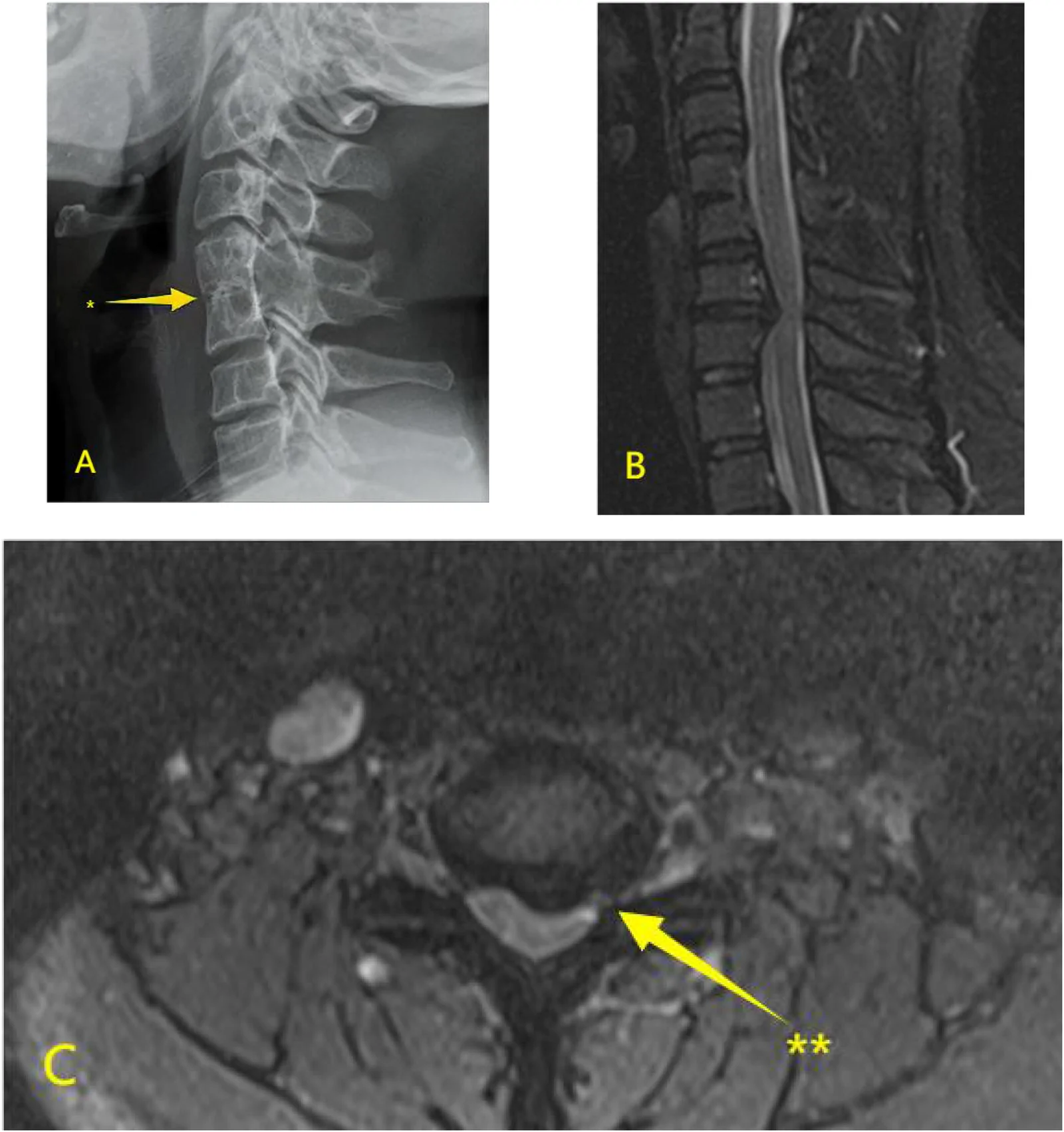
Preoperative cervical imaging. **(A)** Lateral cervical radiograph demonstrating congenital C4/5 vertebral fusion (*), **(B)** Sagittal T2-weighted MRI showing posterior C6/7 disc herniation compressing the spinal cord; **(C)** Axial T2-weighted MRI revealing ventral spinal cord and left nerve root compression (**).

### 2.6 Surgical procedure

After multidisciplinary evaluation and exclusion of surgical contraindications, elective C6/7 anterior cervical discectomy, spinal canal decompression, interbody cage fusion and titanium plate-screw internal fixation were performed.

Intraoperative findings confirmed severe C6/7 disc degeneration and herniation with ventral spinal cord compression. After complete removal of degenerative disc tissue and proliferative osteophytes, thorough decompression of nerve roots and spinal cord was achieved. An 8-mm interbody fusion cage was implanted and fixed with titanium plate and screws. Intraoperative vital signs were stable, and no surgical complications occurred.

## 3 Postoperative outcomes and follow-up

The patient's left upper limb radiating pain was completely relieved immediately after surgery, and sensory and motor functions recovered gradually.

At 12-month postoperative follow-up, neck pain and upper limb numbness disappeared completely, left hand muscle strength recovered to grade 5, and cervical imaging confirmed accurate internal fixation position and solid C6/7 bony fusion (Figure 2).

**Figure 2:**
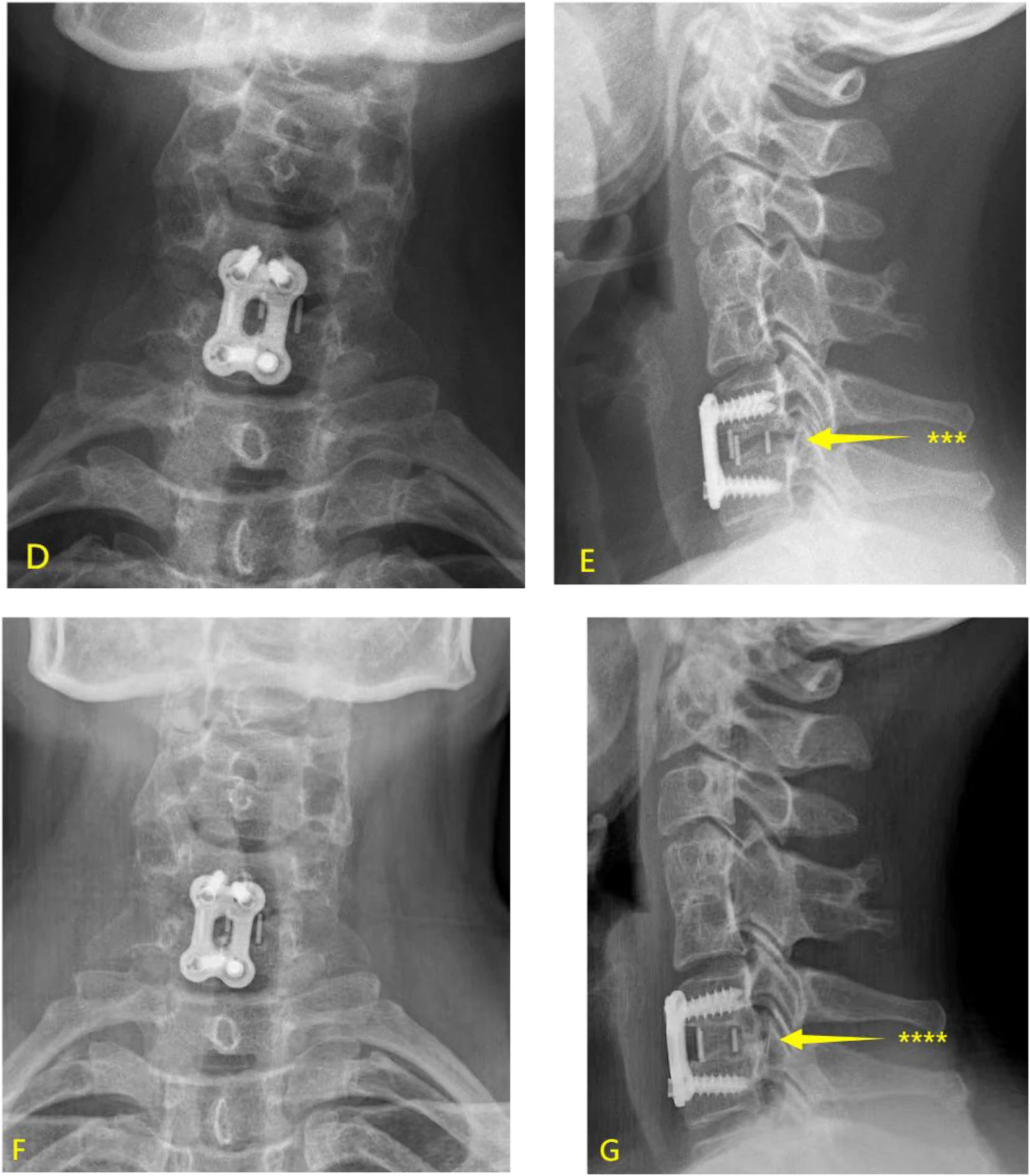
Radiographic follow-up at 12 months after index ACDF. **(D)** Anteroposterior cervical radiograph, **(E)** Lateral cervical radiograph showing appropriate position of interbody cage and titanium plate (***), **(F)** Anteroposterior radiograph confirming solid C6/7 bony fusion, **(G)** Lateral radiograph demonstrating stable internal fixation(****).

At 40 months after index surgery, the patient developed recurrent bilateral upper limb numbness and fatigue without obvious inducement.

Cervical MRI confirmed C5/6 disc herniation and spinal stenosis with nerve compression, which met the diagnostic criteria of postoperative symptomatic ASD (interval ≥12 months after index surgery). After conservative treatment failed again, the patient underwent revision C5/6 ACDF. Revision CDA was not considered appropriate given the patient's congenital fusion deformity and unbalanced cervical biomechanics, which may confer an elevated risk of prosthesis failure. At 6-month follow-up after revision surgery, all clinical symptoms were completely resolved, and cervical CT confirmed stable internal fixation and continuous bony fusion from C4 to C7 (Figures 3, 4).

**Figure 3:**
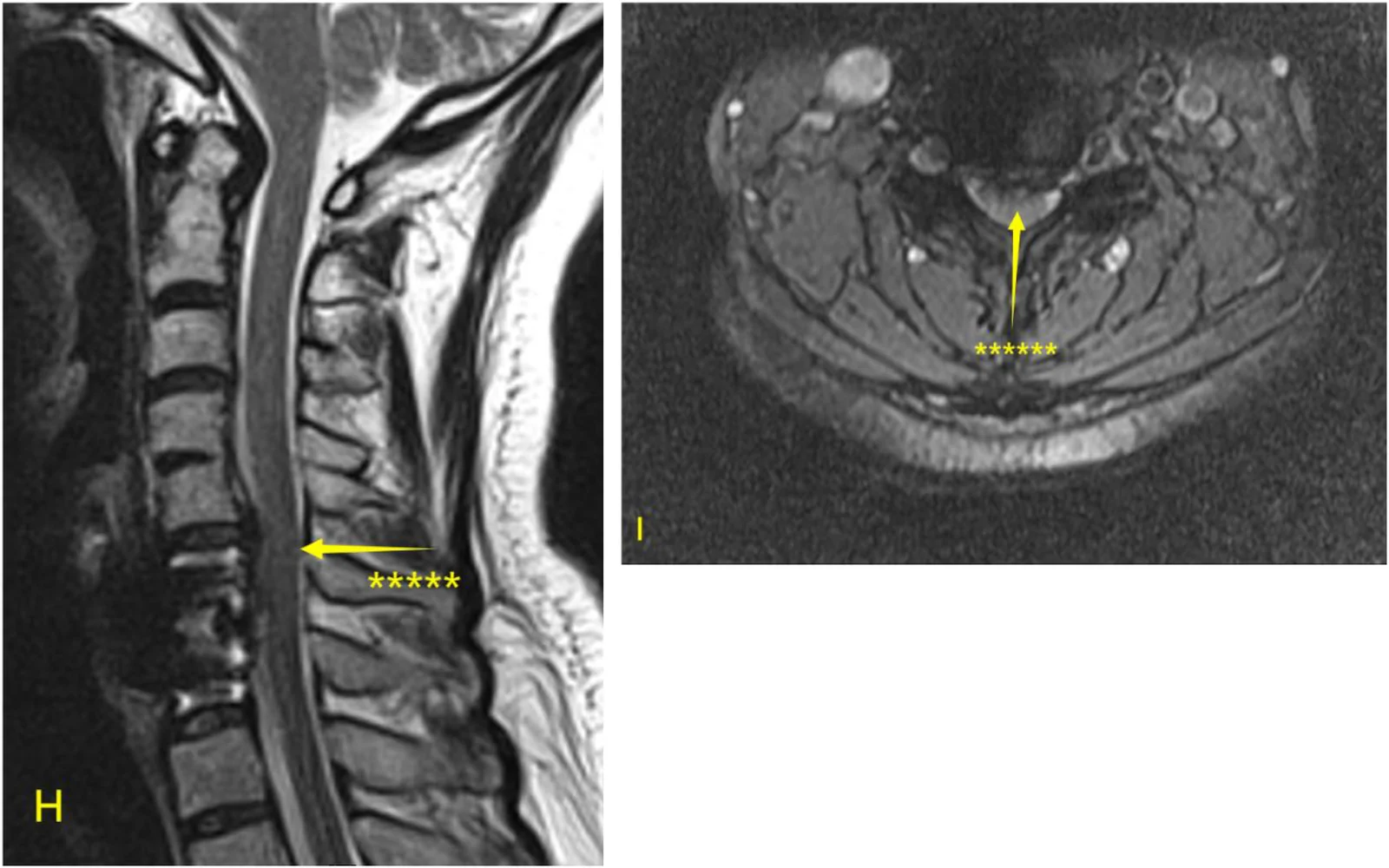
Sagittal **(H)** and axial **(I)** T2-weighted MRI obtained 40 months postoperatively showing C5/6 disc herniation with spinal cord compression (*****, ******).

**Figure 4:**
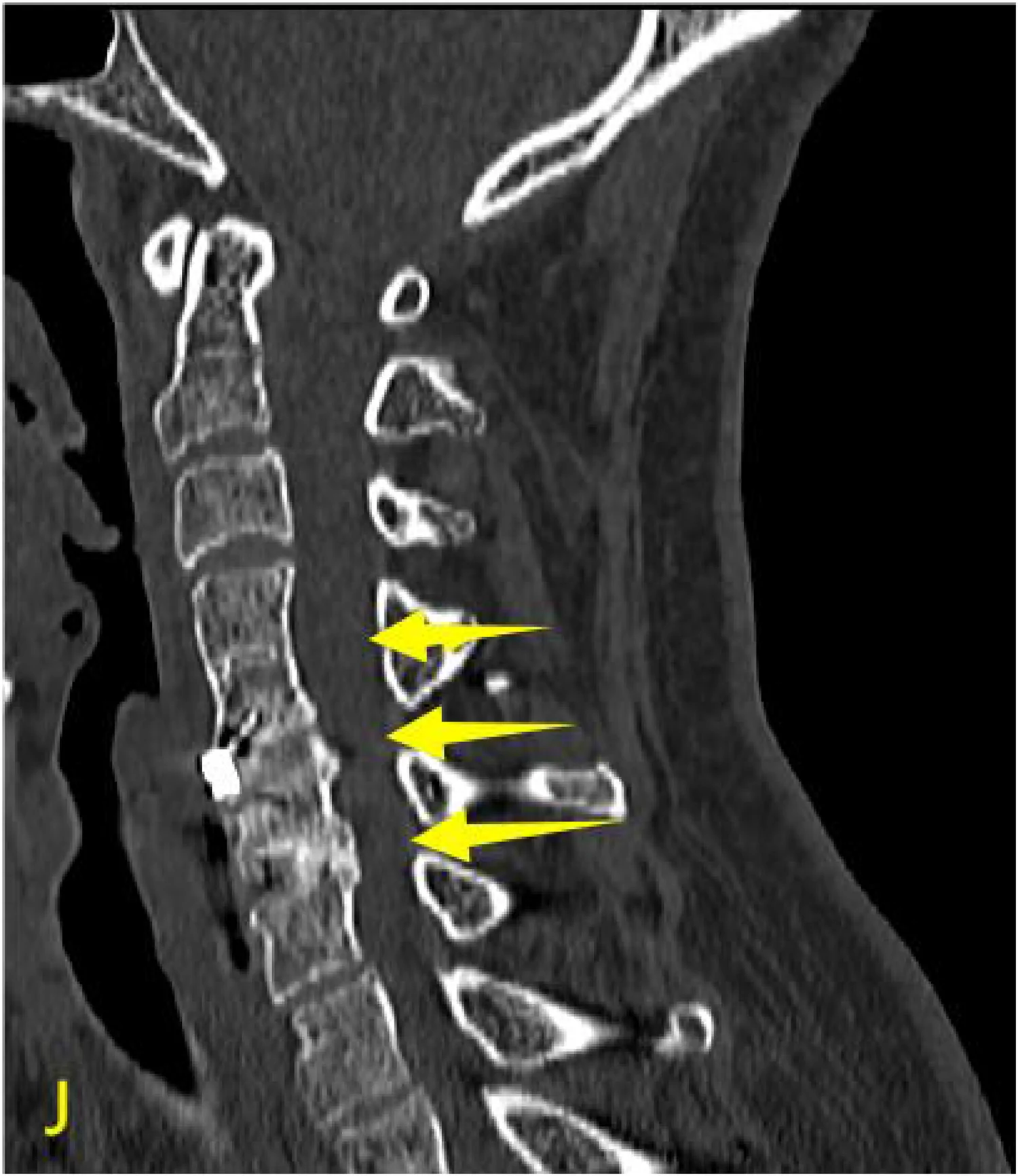
Sagittal CT scan **(J)** at 6 months after revision surgery revealing extensive bony fusion from C4 to C7.

## 4 Discussion

KFS is a heterogeneous congenital spinal malformation with variable clinical manifestations. Most Type I KFS patients have atypical clinical phenotypes, and neurological symptoms are usually secondary to premature cervical degeneration, consistent with the clinical characteristics of the present case (9, 10). Congenital vertebral fusion disrupts normal cervical biomechanical balance, which is the core pathological basis for premature degeneration of residual mobile segments (9).

The core innovation of this case is the rare “sandwich-type interposed mobile segment” biomechanical mode.

The patient retained multiple mobile segments including C0-C1, C1-C2, C2-C3, C3-C4 and C7-T1, but C5/6 was the sole mobile level sandwiched between congenital C4/5 fusion and iatrogenic C6/7 surgical fusion. This unique anatomical structure leads to concentrated shear and rotational stress on the C5/6 level, potentially accelerating degenerative progression. Combined with the 9 systematically retrieved KFS surgical cases, our findings indicate that solitary interposed mobile segments in KFS patients may represent a biomechanically vulnerable segment and may contribute to accelerated early degeneration compared with ordinary cervical segments, and this special biomechanical stress amplification mechanism is rarely reported in previous KFS studies (9, 27).

ACDF is the preferred surgical method for KFS patients with ventral spinal cord and nerve root compression, which can rapidly remove compressive lesions and restore spinal stability (9, 25).

Among the retrieved studies, most KFS patients with single-segment ventral compression achieved favorable short-term outcomes after ACDF, consistent with the present case. However, fusion surgery further increases cervical overall stiffness, aggravating stress concentration of residual mobile segments and increasing long-term ASD risk (13, 14, 30). In contrast, CDA is theoretically superior in preserving segmental mobility, but available evidence regarding CDA in KFS is too limited to draw definitive conclusions. Its long-term effectiveness in patients with abnormal congenital biomechanics remains uncertain, and its ability to prevent ASD in solitary sandwich-type mobile segments has not been validated (22–24).

Notably, single-level primary ACDF was deliberately selected rather than two-stage sequential ACDF or combined anteroposterior surgery during the initial operation, which was based on rigorous preoperative anatomical evaluation, symptomatic characteristics, and standardized surgical indications for Samartzis Type I KFS (8, 9).

First, preoperative imaging confirmed that only the C6/7 segment presented irreversible disc herniation, osteophyte hyperplasia and ventral spinal cord/nerve root compression, while the sandwich-type C5/6 mobile segment showed only physiological stress load without definite degenerative lesions, spinal stenosis or nerve compression (9). Prophylactic multi-level fusion or staged fusion for asymptomatic mobile segments is not recommended in mainstream spinal surgical guidelines and retrieved KFS case evidence, as excessive fusion will further reduce cervical mobility, amplify overall spinal stiffness, and paradoxically aggravate stress concentration on residual intact segments, thereby increasing long-term degenerative risks (16, 25, 32). Second, the patient only presented intractable radicular symptoms induced by C6/7 focal compression, without multi-segmental neurological deficits, cervical dynamic instability, or posterior osseous canal stenosis that required posterior decompression. Combined anteroposterior surgery is mainly indicated for KFS patients with complex multi-level compression, severe spinal deformity, kyphotic malalignment, or posterior element hyperplastic stenosis, which was inconsistent with the patient's single-level focal lesion characteristics (28, 29). Third, two-stage ACDF is primarily applied for severe multi-segmental cervical degeneration with staged neurological compression or insufficient single-stage decompression, which was unnecessary for this patient with localized single-level symptomatic lesion. According to the included KFS surgical cases, excessive prophylactic fusion or combined surgery for mild single-segment KFS lesions cannot reduce ASD incidence, but significantly increases surgical trauma, bleeding volume, and postoperative recovery burden (16, 31). Therefore, individualized single-level ACDF was the most rational and minimally invasive surgical strategy for the primary operation, adhering to the principle of “targeted decompression, limited fusion, and maximum preservation of physiological spinal function” for congenital cervical malformation surgery.

Literature review showed that young age (≤50 years), preoperative adjacent segment degeneration, and non-traumatic surgical indications are independent risk factors for postoperative ASD reoperation (14, 27).

The 44-year-old patient presented with multiple superimposed high-risk factors, including younger age and the unique sandwich-type anatomical malformation, which collectively accounted for the occurrence of early symptomatic ASD at 40 months after the index surgery. In addition, rare postoperative complications such as white cord syndrome and abnormal bony fusion malformations have also been reported in cases of cervical surgery performed on patients with KFS (26, 27). This suggests that cervical surgery in KFS patients is associated with higher intraoperative and postoperative risks compared with that in the general patient population (17, 18, 21), thus necessitating precise preoperative evaluation and intensive postoperative monitoring (26, 31). For KFS patients with concomitant Sprengel deformity and vertebral bony malformations, individualized decompression strategies should be developed based on their abnormal anatomical compression characteristics (19, 20).

### 4.1 Strengths and limitations

Strengths: (1) This case first reports the early ASD characteristics of Samartzis Type I KFS with sandwich-type interposed mobile segment, supplementing scarce biomechanical clinical evidence; (2) Standardized CARE-compliant clinical timeline and complete multi-stage imaging data ensure the integrity and standardization of case data; (3) Combined with dual-database retrieved 9 KFS surgical cases, the study systematically summarized the surgical modality selection and long-term complication risk of KFS, enhancing the academic rigor and clinical reference value of the single case.

Limitations: (1) As a single-case report, it cannot establish a definite causal relationship between sandwich-type biomechanics and early ASD, which needs large-sample cohort studies for further verification; (2) Revision surgery was performed in an external hospital, lacking detailed intraoperative records; (3) Long-term follow-up data after revision surgery remain limited; (4) Targeted large-sample epidemiological data for KFS ASD are still lacking, and further accumulation of clinical cases is required.

## 5 Conclusion

ACDF is safe and effective for the treatment of Samartzis Type I KFS complicated by cervical radiculopathy and radiographic spinal cord compression, achieving reliable short-term neurological functional recovery (9, 25).

However, KFS patients with unique sandwich-type interposed mobile segment biomechanical structure may have an increased risk of early symptomatic adjacent segment degeneration. Based on Web of Science and PubMed dual-database systematic literature screening and case summary, this study further suggests that KFS patients have individualized degenerative risk characteristics different from ordinary cervical spondylosis patients (13–15). Precise preoperative biomechanical evaluation, individualized surgical strategy formulation, and lifelong regular imaging surveillance are essential to reduce long-term postoperative complications and improve long-term prognosis of KFS patients (9, 12).

## Data Availability

The original contributions presented in the study are included in the article/Supplementary Material, further inquiries can be directed to the corresponding author.
